# Report of the County Medical Officer of Health and School Medical Officer (L.C.C.) for 1915

**Published:** 1916-10-14

**Authors:** 


### THE HEALTH OF LONDON.

### Report of the County Medical Officer of Health and School Medical Officer (L.C.C.) for 1915.

Statistical tables and percentages and the relations of these to other tables and percentages do not make lively reading, and the cunning which can tempt the man in the street to study them is not usually a quality of the official mind. There are many industrious attempts in this direction, but the hand of the magician is not a frequent appearance. Where, however, the public health and the national life are in question it is facts which are the essential thing, for these form the stuff out of which wise policy and enlightened administration may be evolved. More than once it has been suggested in these columns that an attempt might be made to give in some broad and picturesque form the general truths which emerge from the facts and figures enshrined in public health reports. Only by some such method can the mass of the people be led to understand and to appreciate the nature of the services which secure their protection from omnipresent risks and dangers. The reports themselves are too ponderous, too detailed, and too rigid for popular consumption. Not that we condemn them, or question their necessity. But we suggest that their teaching could be brought to touch more nearly the mass of the people. Too many of us enjoy all the advantages of sanitary protection without giving a thought to the machinery by which these advantages are secured.

### The Dangers of Shaving.

Dr. Hamer's record for 1915 in dealing with the Administrative County of London is necessarily one of the most important of public-health documents, and we may extract from it a few experiences which illustrate the protective value of the machinery which acts under the medical officer's direction. Here, as elsewhere, the war exercises its influence. That it should have cut down the working staff of medical officers, of sanitary inspectors, and of administrators was to be expected. But who would have anticipated that among the consequences of the war must be numbered the risk of a serious outbreak of anthrax in the British Isles? Yet the facts show this to be abundantly true, and indeed safety was only secured by vigour and decision resting on skilled scientific knowledge. The medium of distribution was the shaving-brush, and though this has often been accused more or less vaguely of many possible ills, never before has bacteriological demonstration shown it to harbour the anthrax bacillus. That the brush may retain this organism in a potentially active state in spite of the various manufacturing processes to which the hair is subjected, is a disconcerting experience, and apprehension becomes still more acute on learning that some 1,500 of such brushes have gained admission to this country. Many of them fortunately have been identified and recovered, but there are still some which have not been traced. The influence of the war in this affair is interesting. In less disturbed days it seems that "hair" from China is imported into Switzerland and thence sent to Germany, where it is cut into lengths and disinfected. It then returns to Switzerland, where it is made into brushes, and these are sold to dealers in this country. But this peaceful course of events has been disturbed, and thus it comes about that the "hair" came from China direct to England, and as it was labelled "goats' hair" (a lie of the label) it escaped the Home Office regulations relative to disinfection. Hence seven cases of anthrax with two deaths and a risk to large numbers of the men who cultivate clean chins. The illustration is an impressive one of the practical demands made on those whose duty it is to guard the national well-being. Rules and regulations have their value, but, as experience repeatedly shows, the price of epidemic safety is eternal vigilance. Of a less dramatic order, because custom dulls its appeal, is the record of such diseases as smallpox and typhus. It will surprise some of our readers to learn that in 1915 there were four importations of smallpox into London. Yet the spread of the disease was limited to some ten cases. Again, typhus made its appearance in the same year, but only four patients were involved in the outbreak.

### King Edward's Conundrum.

If victory seems assured over some of the scourges of mankind there still remain evils enough for our undoing. Measles, scarlet fever, diphtheria, enteritis, and whooping-cough continue to exact a heavy toll of human life, and more especially of life in its early days, while tubercle as ever spares neither age nor sex. Yet all these fall among the "preventable" diseases, and recall the question originally presented by the late King Edward, "Why, then, not prevented?" The circumstances of the hour emphasise the terrible figures of infant and child mortality, and patriotism not less than humanity calls for some serious efforts to stay the mischief. If Public Health has reason to be proud of its record it is still very far from a complete triumph. Some part of its failure is due to want of money. Let the people be informed of the issues which are at stake and money will certainly be forthcoming. Experience is teaching us large lessons in national expenditure, and not only men but purses have their patriotic obligations. A study of such reports as the one now before us suggests social conclusions as well as medical ones. The most urgent need of the moment seems to be men in the vigour of early manhood and fit to stand in the fighting-line. In so far as we are poor in these is the deficiency not largely due to the national apathy towards questions of public health and the protection of infancy and childhood? Viewed in this light the work of the medical officer is seen to be of the highest national importance, and it must not be hindered by individuals.

